# MiR-155: An Important Regulator of Neuroinflammation

**DOI:** 10.3390/ijms23010090

**Published:** 2021-12-22

**Authors:** Valeria Domenica Zingale, Agnese Gugliandolo, Emanuela Mazzon

**Affiliations:** IRCCS Centro Neurolesi “Bonino-Pulejo”, Via Provinciale Palermo, Contrada Casazza, 98124 Messina, Italy; valeria.zingale@irccsme.it (V.D.Z.); emanuela.mazzon@irccsme.it (E.M.)

**Keywords:** miR-155, neuroinflammation, microRNAs, neurodegenerative disease, multiple sclerosis, Alzheimer’s disease, Parkinson’s disease, ischemic stroke, amyotrophic lateral sclerosis

## Abstract

MicroRNAs (miRNAs) are small non-coding RNA molecules that regulate gene expression at the post-transcriptional level and that play an important role in many cellular processes, including modulation of inflammation. MiRNAs are present in high concentrations in the central nervous system (CNS) and are spatially and temporally expressed in a specific way. Therefore, an imbalance in the expression pattern of these small molecules can be involved in the development of neurological diseases. Generally, CNS responds to damage or disease through the activation of an inflammatory response, but many neurological disorders are characterized by uncontrolled neuroinflammation. Many studies support the involvement of miRNAs in the activation or inhibition of inflammatory signaling and in the promotion of uncontrolled neuroinflammation with pathological consequences. MiR-155 is a pro-inflammatory mediator of the CNS and plays an important regulatory role. The purpose of this review is to summarize how miR-155 is regulated and the pathological consequences of its deregulation during neuroinflammatory disorders, including multiple sclerosis, Alzheimer’s disease and other neuroinflammatory disorders. Modulation of miRNAs’ expression could be used as a therapeutic strategy in the treatment of pathological neuroinflammation.

## 1. Introduction

MicroRNAs (miRNAs) are a large group of small endogenous molecules of single-strand non-coding RNA, approximately 21–25 nucleotides in length, and their fundamental role is to regulate gene expression at the post-transcriptional level. They act as negative regulators by binding through a specific region (seed) the 3′ UTR of a mRNA target and determining its degradation or the inhibition of its translation [1]. 

The first miRNA was discovered by studying the development of nematode *Caenorhabditis elegans* with the identification of the developmental regulator LIN-4 [2]. In 2000, the second miRNA let-7 was discovered in *C. elegans*, and it was found to be preserved in many organisms, including humans, suggesting that this class of small regulatory RNA has a more general role in biology [3].

Gene-expression regulation mediated by miRNAs seems to affect more than 30% of human genes. It has been shown that each miRNA may regulate different mRNA targets and that multiple miRNAs may regulate the same mRNA. This allows us to consider miRNAs as elements of control of numerous pathways, which regulate fundamental cellular processes, such as cell cycle regulation, cell proliferation, differentiation and apoptosis [4]. It has been shown that alterations in miRNAs expression may be involved in the onset and progression of several diseases, such as cancer, cardiovascular and neurodegenerative diseases [5]. Various studies have documented the importance of miRNAs in inflammatory conditions and in various central nervous system (CNS) pathological conditions, including neuroinflammation, neurodegeneration and autoimmune diseases [6]. Given the importance of immune-inflammatory processes in neurological diseases, research in recent years has focused on understanding the mechanisms involved and possible therapeutic targets. Numerous evidences have shown the involvement of miR-155 in the neuroinflammatory signaling and in the intensification of the neurological damage. This review focuses on the role of miR-155 in the pathogenesis and progression of several neurological disorders, including multiple sclerosis (MS), Alzheimer’s disease (AD) and other neuroinflammatory conditions.

## 2. MiRNAs

The gene-encoding miRNAs are transcribed by RNA polymerase II, and only in some cases by RNA polymerase III [7].

A primary miRNA, the pri-miRNA, is produced in the cell nucleus, where it assumes a double-stranded spiral conformation. Subsequently, Drosha, a Rnase of 160 kDa with endonuclease activity, in cooperation with the factor DGCR8 (Di George Syndrome Critical Region Gene 8), binds to the stem–loop structure of the pri-miRNA and makes site-specific cuts, leading to pre-miRNA release. This step is called cropping. The pre-miRNA, which is about 60–80 nucleotides long, has a loop and a double-stranded stem of about 33 nucleotides complementary to each other and is characterized by two or three nucleotides protruding at 3′ and a phosphate group at 5′. Afterward, the pre-miRNA is exported to the cytoplasm by Exportin-5 to continue its maturation process [8].

In the cytoplasm, the Dicer enzyme, with the TRBP (TAR RNA binding protein) complex, cleaves to the pre-miRNA to create a duplex RNA molecule of 18–25 nucleotides; subsequently, the miRNA duplex activates the complex RISC (RNA-Induced Silencing Complex) and the Argonaute 2 protein (AGO-2), which will direct the miRNA to recognize the mRNA target [9]. Among the two strands constituting the duplex, the one that has lower thermodynamic stability at the 5′ end will be selected as a guide strand, complementary to the target messenger, while its complement will be degraded. When the two ends 5′ have comparable stability, each of the two strands with the same probability can go on to make the ripe miRNA. In this regard, in the nomenclature a tag is added, indicating from which filament the mature miRNA was derived: -5p is used if it comes from the 5′ precursor, and -3p is used if instead it derives from the 3′ of the precursor. The mRNA target is recognized by sequence complementarity with the miRNA seed sequence, consisting of 2–8 nucleotides, present at the 5′-end of the guide strand [10]. 

Based on complementarity between miRNA and mRNA target, RISC complex may inhibit mRNA expression through two different processes:

- Degradation of the messenger, which occurs in the case of perfect complementarity between the two sequences [11].
- Inhibition of translation, that occurs in the presence of mismatches between the two sequences [12].

MiRNAs are present in high concentrations in the CNS and are expressed in a spatially and temporally specific way; the gene silencing mediated by these small molecules seems to be fundamental in all the stages concerning the development of brain and the maintenance of homeostasis. Post-transcriptional regulation mediated by miRNAs modulates processes such as neurogenesis, neuronal differentiation, synaptic plasticity, gliogenesis and myelin repair; therefore, an imbalance in the expression pattern of these small molecules can be involved in neurological pathologies [13], abnormalities of neurological development, neurodegenerative and autoimmune processes [14].

Numerous evidences support the involvement of miRNAs in the modulation of inflammatory signaling within the CNS. Depending on their activity, miRNAs can promote or reduce inflammatory signaling, and in conditions of uncontrolled neuroinflammation, they can aggravate or improve pathological consequences [15]. Then the neuroinflammatory response is the result of the synergistic or antagonistic activity of several miRNAs with pro-inflammatory action (e.g., miR-155, miR-27b and miR-326) and anti-inflammatory activity (e.g., miR-124, miR-146a, miR-21 and miR-223) or by mixed immunomodulators, such as let-7 family [16].

The miRNAs in the neuroinflammatory process can regulate the activation of microglia and astrocytes and also control the activity of peripheral immune cells, such as neutrophils, macrophages, leukocytes and T and B cells [17].

In the context of neuroinflammation, the regulation modes of several miRNAs have been analyzed; among the most important are miR-155, miR-146a, miR-124, miR-21 and let-7 [15]. Some miRNAs modulators of inflammation were evaluated in microglia cultures, demonstrating their role in the inflammatory activation of microglia in CNS. In particular, miR-689, miR-124 and miR-155 were the most strongly associated with pro-inflammatory signaling and microglia activation phenotype. After exposure to lipopolysaccharide (LPS), cultured microglia showed increased expression of miR-155, but reduced expression of miR-689 and miR-124 [18].

Recent studies have revealed that miRNAs are involved in the differentiation of T and B cells, in the signaling of transduction by Toll-like receptors (TLRs) and in the production of cytokines. For example, the expression of miR-146a, miR-155 and miR-132 is altered in response to LPS, Tumor Necrosis Factor (TNF-α), and interleukin (IL)-1β. It has been observed that miR-146 plays an anti-inflammatory role in reducing excessive inflammation, while miR-155 plays a pro-inflammatory role in regulating inflammatory cytokines, such as interferon (IFN)-λ and IFN-β [19]. Their deregulated expression can lead to the uncontrolled proliferation of immune cells and the activation of inflammatory pathways, leading to the development of pathological processes [20].

## 3. Neuroinflammation

The term “neuroinflammation” refers to an inflammatory response within the CNS, with the aim of promoting cellular homeostasis in physiological and pathological conditions [21].

Under physiological conditions, the CNS produces pro-inflammatory factors in response to different injuries. In this case, the activation of inflammatory process has a neuroprotective effect and promotes tissue repair [22].

However, in pathological conditions, uncontrolled neuroinflammation can cause tissue damage and become a major component of many neurodegenerative diseases. In fact, the uncontrolled activation of inflammatory processes determine an excessive activation of glial cells, leading to the production of pro-inflammatory cytokines, such as IL-1β, IL-6 and TNF-α; chemokines; secondary messengers, including nitric oxide (NO); prostaglandins; and reactive oxygen species (ROS) [23,24]. In addition, the activation of inflammatory processes cause the loss of the blood–brain barrier (BBB) and the infiltration of peripheral immune cells [25].

In response to injury, the inflammatory response is activated by glial cells residing in the CNS, particularly microglia and astrocytes. 

Microglial activation provides the first line of defense when injuries or diseases occurs. Under normal conditions, microglia are in a “quiescent” or “resting” state, where they actively supervise the microenvironment and help maintain cerebral homeostasis [26]. The activation of microglia occurs in the presence of infections and tissue damage and is accompanied by several morphological changes; in fact, they pass from a branched to an amoeboid morphology that allows motility and phagocytosis. Microglia can attack healthy neurons through phagocytosis or secretion of apoptotic factors [23]. Depending on the nature of the signals, activated microglia can differentiate into classical phenotype M1 (pro-inflammatory) or alternative phenotype M2 (anti-inflammatory) [27]. M2 microglia release anti-inflammatory and protective cytokines, such as Transforming Growth Factor β (TGF-β), IL-10, IL-4 and IL-13, which play important roles in wound healing and tissue repair. Conversely, M1 microglia releases an excess of inflammatory mediators, such as ROS, superoxide anion, matrix metalloproteinase (MMP)-9 and pro-inflammatory cytokines, such as TNF-α, IL-6 and IL-1β [28]. Microglia activation and the resulting morphological and functional changes have been observed during almost all neuropathological conditions, including neurodegenerative diseases, infections, strokes, tumors and brain injuries [29]. As a result of uncontrolled processes of neuroinflammation, these cells can remain active for long periods, contributing to neurodegeneration through the continuous release of cytokines and neurotoxic molecules. Inhibition of pro-inflammatory mediators produced by microglial cells could be considered a valid therapeutic approach to reduce the progression of neurodegenerative diseases [30]. 

Astrocytes are another type of glial cells that play an important role in influencing the inflammatory response of the CNS in case of stress or diseases [31]. Under normal conditions, astrocytes help to maintain homeostasis through interactions with the neuronal signaling system, providing metabolic support, regulating synaptogenesis and through the clearance of neurotransmitters. They also regulate the extracellular space volume and modulate the synaptic plasticity [32]. Astrocytes can promote or reduce neuroinflammation through the release of pro-inflammatory and anti-inflammatory molecules, and acting as functional barriers to the CNS parenchyma [33]. Astrocytes and microglia express different types of receptors, including TLRs, whose activation triggers the neuroinflammatory reaction [34]. Among these, of particular importance is the TLR4, which is considered a key receptor of pro-inflammatory signaling. Its activation induces the release of TNF-α and IL-1β, which activate the inflammatory signaling cascade [30].

Endothelial cells and peripheral immune cells also play a role in the propagation of these inflammatory signals [21]. Neuroinflammation is also regulated through complex signaling cascades between different cell types within the CNS, such as the neurovascular unit (NVU). The NVU is a structure composed of neurons, astrocytes, extracellular matrix and the microvascular endothelial brain, which play a key role in controlling the neuroinflammatory process [35]. This network allows intercommunication between blood vessels and neurons within the CNS and allows regulation of blood flow, brain development, BBB permeability, elimination of toxic by-products and immune surveillance [36].

Thus, neuroinflammation is mediated through the complex interaction between CNS cells and peripheral cells. Understanding the cellular and molecular mechanisms that control neuroinflammation is a field of research that has generated much interest in the discovery of new therapeutic approaches. Among the central regulators of these processes, there are miRNAs, which, if deregulated, may contribute to the progression of the disease, or may reflect a homeostatic attempt of the CNS to prevent and restore normal conditions [15]. Their role in initiating and maintaining neuroinflammation is well reported.

## 4. MiR-155

MiR-155 is encoded by the MIR-155 host gene, *mir155hg*, also called the B-cell Integration Cluster (BIC) gene [37]. The BIC gene is composed of three exons within a 13 kb region located in human chromosome 21q21 and produces a pri-miR-155 of 1500 bp transcript in exon 3, which is processed to the mature miR-155. Based on the processing of pre-miR-155 (65 bp), the mature sequence of miR-155-5p or miR-155-3p is obtained [38,39].

The involvement of miR-155 in inflammatory processes was first suggested in a study of human B-cell lymphoma where its expression was found to be significantly elevated [40].

MiR-155 is highly conserved and plays an important role in the immune system of mammals; numerous studies support the involvement of miR-155 in regulating the differentiation of helper T cells and in the regulation of the response by macrophages through the production of cytokines [41].

In mouse macrophages knockout (KO) for miR-155, a reduction in inflammatory signaling was observed. In addition, there was a 72% reduction in the activity of the inflammatory genes iNOS (inducible nitric oxide synthase), IL-1β and TNF-α after stimulation with IFN-γ + LPS. These data support the role of miR-155 in inflammatory macrophage signaling [42].

Later studies have shown the presence of miR-155 in numerous tissues, including the brain, confirming the pro-inflammatory action of this miRNA in both the peripheral immune system and the CNS [43].

MiR-155 is considered a pro-inflammatory mediator of the CNS and results upregulated in the brain of patients affected by many neurodegenerative diseases. Its action is induced in microglia and macrophages through the nuclear factor κB (NF-κB), after stimulation of the TLR and release of the pro-inflammatory cytokine IFN-γ [19,44,45].

MiR-155 induces neuroinflammation through the inhibition of factors involved in the inflammatory process. The involvement of miR-155 determines a reduction of endogenous anti-inflammatory response resulting in increased inflammation. Some targets of miR-155 include anti-inflammatory regulators, such as Suppressor of Cytokine Signaling (SOCS1), a negative regulator of cytokines [43]; SH2 Domain-Containing Inositol 5′-Phosphatase1 (SHIP1), a negative regulator of TNF-α [46]; and IL-13 receptor alpha 1 (IL13Rα1) [47].

Cardoso et al. showed the pro-inflammatory role of miR-155 following microglia activation, suggesting that post-transcriptional modulation of SOCS-1 determines the progression of the immune response. SOCS-1 is part of a group of proteins inhibiting the cytokine signal translation pathways. In fact, it plays an important role in regulating immune response through direct inhibition of Janus tyrosine kinase (JAK) and consequent inhibition of signal transducer and transcription factor activator (STAT). In vitro studies in N9 microglia cells exposed to LPS evidenced the upregulation of miR-155 and the reduction in SOCS-1 levels. In addition, anti-miR-155 oligonucleotides decreased the expression of inflammatory cytokines IL-6, IFN-β and TNF-α and the production of NO with subsequent reduction of neuronal death. These results suggest that miR-155 inhibition induces neuronal protection and miR-155 could be a target for controlling neuronal inflammation [43]. 

In addition, miR-155 is involved in the gene regulation of astrocytes. Activation of these cells induces upregulation of miR-155, which, by inhibiting the mRNA of SOCS-1, determines the high production of pro-inflammatory cytokines [48].

Stimulation of RAW264.7 and THP-1 cells with LPS results in increased miR-155 expression levels correlated with SOCS-1 repression and increased TNF-α and IL-6 production. Treatment with curcumin significantly reduced miR-155 levels and cytokine production, indicating miR-155 as a potential target for suppressing inflammatory responses. Curcumin inhibits miR-155, TNF-α and IL-6 through the PI3K-AKT pathway, suggesting a crucial role of this pathway in the regulation of miR-155 [49].

MiR-155 was one of the first miRNAs to be related to the activation processes of the phenotype M1 of microglia. Resting microglia cells are characterized by low levels of miR-155 expression. On the other hand, in the presence of strong inflammatory stimuli, microglia assume an M1 phenotype, characterized by high levels of miR-155 expression. Upregulation of miR-155 is believed to be crucial for the establishment of this phenotype, since this miRNA acts directly on anti-inflammatory molecules, such as SOCS-1, leading to over-regulation of the various inflammatory mediators characteristic of the M1 phenotype [50].

In microglia, miR-155 also acts through the involvement of the transcriptional factor p53 (tumor protein P53), and c-Maf (musculoaponeurotic fibrosarcoma). Moreover, p53 is activated in microglia by ROS, DNA damage or cell stress associated with CNS disease and injury and promotes the expression of the BIC gene coding for miR-155. MiR-155 degrades c-Maf, known for its anti-inflammatory activity, thus promoting pro-inflammatory processes of microglia. The correlation between p53, miR-155 and c-Maf was analyzed in adult murine microglia after induced neuroinflammation through the middle cerebral artery occlusion (MCAO) for 15 min. Two more miRNAs, miR-34a and miR-145, were considered. Activation of p53 induces miR-155, miR-145 and miR-34a. While miR-155 acts on the c-Maf factor, miR-145 and miR-34a act on Twist2 (Twist Family BHLH Transcription Factor 2), an activator of the c-Maf expression. Both pathways downregulate the expression of the c-Maf anti-inflammatory transcription factor [51].

### 4.1. MiR-155 and Alzheimer’s Disease

AD is an aging-associated progressive neurodegenerative condition characterized by memory loss, impaired cognitive functions and behavioral changes [52]. It is characterized by the presence of senile plaques caused by extracellular deposits of beta-amyloid peptide (Aβ) and neurofibrillar tangles consisting of accumulations of the hyperphosphorylated protein Tau [53].

Neuroinflammation plays an important role in the pathogenesis and progression of AD. Increased microglia activation has been observed, resulting in the release of inflammatory mediators and progressive neuronal degeneration [54]. The release of ROS by microglia activates the NF-κB dependent signaling pathway, which amplifies the inflammatory response through the production of a large number of inflammatory factors. In addition, activation of the NF-κB pathway induces upregulation of the site 1 amyloid precursor protein splitting enzyme (BACE1) and promotes the production of large quantities of Aβ [55].

Experimental studies concerning miR-155 in AD are summarized in Table 1.

Many studies have focused on the aggregation of Aβ, in particular, Aβ42, which seems to be the main cause of AD development. In AD, the peptide Aβ42 is present in high quantities and initiates the mechanism of polymerization by forming neurotoxic lamellar structures [56]. 

An in vitro study on LPS-stimulated microglia cells showed the overexpression of miR-155 related to a reduction in Aβ42 catabolism. These data support the involvement of inflammatory processes modulated by miR-155 on the activity of microglia to catabolize Aβ42 [57].

High levels of miR-155 were shown in blood-derived monocytes (BDMs) and monocyte-derived macrophages (MDMs) isolated from AD patients. The correlation between upregulation of this miRNA, BDMs M1 activation status and reduction of Aβ phagocytosis was highlighted [58].

Lukiw et al. underlined the increased presence of miR-155 in neocortical extracellular fluid (ECF) and cerebrospinal fluid (CSF) of AD patients. Subsequently, they showed that miR-155 was secreted by human co-cultures of neuronal-glia (HNG) stressed by TNF-α and Aβ42 peptide. Finally, they demonstrated that a conditioned medium (CM) containing miR-155 induced inflammatory gene expression in control HNG cells [59]. In addition, HNG cultures stressed with AD-derived ECF showed increased regulation of some miRNAs, including miR-155 [60].

MiR-155 upregulation has been shown in mice APPtg and TAUtg and in SAMP8 mice, an accelerated aging animal model with behavioral and histopathological characteristics of AD. Having as targets many key genes of the pathological process of AD, the deregulation of miR-155 could cause deficits in cellular functions and alterations in the biological processes involved in AD [61,62]. In particular, the enrichment analysis of gene ontology (GO) categories showed a significant enrichment of predicted targets in terms of regulation of neurogenesis, regulation of the development of dendritic spines, regulation of immune system process, T-cell activation, T-cell differentiation and the regulation of myeloid cell differentiation [62].

Recent studies highlighted the role of miR-155 in regulating survival, differentiation, proliferation and activation of T cells during inflammatory signaling in AD. In particular, it seems to be associated with the interaction between dendritic cells and T cells; in the regulation of differentiation and proliferation of T cells Th17, CD4^+^, Th1, Th2 and CD8^+^; and in the survival of Treg cells [67]. 

Guedes et al. supported the involvement of miR-155 in neuroinflammation associated with AD. In this study, increased levels of miR-155 in the animal model 3xTg AD were indicated simultaneously with increased activation of microglia and astrocytes and downregulation of SOCS-1. In addition, the interaction of miR-155 with the c-Jun terminal transcription factor of the JNK pathway was highlighted, together with their role in the production of inflammatory mediators, such as IL-6 and IFN-β. To demonstrate the possible involvement of c-Jun in miR-155 overexpression after microglia and astrocytes activation by Aβ, N9 microglia cells and primary astrocyte cultures were treated with a siRNA targeted at c-Jun (siRNA c-Jun). A significant reduction in the expression of miR-155 has been observed. These results were confirmed by in situ hybridization studies, which revealed a significant reduction in the marking of miR-155 in cell cultures [63]. 

Although further studies on the role of miR-155 in the development and progression of AD are needed, some evidence identified miR-155 as a potential therapeutic target. It has been shown that the upregulation of miR-155 in AD is reduced through the use of anti-NF-kB agents or substances with anti-inflammatory properties. For example, miR-155 upregulation was reduced by using anti-NF-kB agents such as caffeic acid phenethyl ester (CAPE) and the potent resveratrol analog 1-fluoro- 2-[2-(4-methoxyphenyl)-ethenyl]-benzene (CAY10512) [60]. In apoE3-5xFAD mice, treatment with curcumin significantly reduced miR-155 expression levels in correlation with a reduction in the neurodegenerative microglial phenotype [64]. The action of other anti-inflammatory molecules on miR-155 expression has been investigated, such as hydrogen sulfide (H_2_S) and microvesicles extracted from mesenchymal stem cells (MSC). AD was induced in albino Wistar rats by intraperitoneal injection of LPS; monotherapy treatment reduced miR-155 levels and inflammation, decreased apoptosis and improved histopathological alterations of the hippocampus [65].

Neuroinflammation also affects cognitive functions in the pathophysiological process of AD. In an AD rat model, the effects of miR-155 on the expression of IL-1β, IL-6 and TNF-α were determined in the hippocampus. Treatment with an anti-miR-155 reduced the upregulation of these factors and attenuated the increase of Caspase-3, resulting in the recovery of learning functions that were compromised [66].

In AD the upregulation of miR-155 also leads to the downregulation of the complement factor H (CFH), a complement glycoprotein that has the function of suppressing the immune response. The downregulation of CFH activates the complement system during the propagation of neuroinflammation and would appear to have a critical role in the regulation of immune response and in the onset and development of neurodegeneration [68].

### 4.2. MiR-155 and Multiple Sclerosis

Multiple sclerosis (MS) is an autoimmune demyelinating disease characterized by damage to the myelin sheath surrounds nerve cells. It is characterized by disorders of sensory, motor and cognitive function, with symptoms of pain and fatigue [69].

Different subtypes of MS can be distinguished. Relapsing–remitting multiple sclerosis (RRMS) is characterized by alternate periods of attacks and remission. In most cases, this form advances to a subtype of secondary progressive multiple sclerosis (SPMS). Primary progressive MS (PPMS) is characterized by an initial attack with a constant worsening of the disability [70].

The pathophysiology of MS is based on the involvement of immune cells, such as CD4^+^ T cells, CD8^+^ T cells and B cells, which cause a persistent inflammatory state. During the development of MS, the BBB becomes dysfunctional and allows these cell types to infiltrate the brain [71]. The malfunction of this protection barrier is due to the activity of pro-inflammatory cytokines, such as TNF-α and IFN-γ [72].

MiR-155 has been identified as one of the most important miRNAs in MS. An overview of experimental studies on miR-155 in MS is given in Table 2.

Overexpression of miR-155 was observed in a number of samples, including active brain lesions and blood cells of MS patients and in vivo studies in the experimental autoimmune encephalomyelitis (EAE) animal model [86].

Through a profiling of the miRNAs differentially expressed in serum and plasma of patients with MS and a subsequent computational analysis, it was found that miR-155 has as targets PIK3R1 and PIK3CA, which are considered to be MS risk genes. These genes encode proteins belonging to the family of phosphoinositide 3-kinase (PI3K). Studies show that the abnormal expression of PI3K contributes, for example, to neurological and immunological dysfunctions, to the pathogenesis of EAE and to the demyelination in MS [73]. In a study conducted on peripheral blood mononuclear cells (PBMC) of MS patients, the expression levels of 22 miRNAs related to immunity were analyzed, and miR-155 was found to be the most upregulated. In addition, miR-155 was studied by genotyping four single nucleotide polymorphisms (SNPs) in the miR-155 genomic region, and the results obtained showed its involvement in the regulation of MS pathophysiology. A haplotype was over-represented in MS cases compared to controls, thus resulting in the conclusion that it is associated with the disease status. This haplotype conferred a 1.36-fold increased genetic risk of developing MS, again supporting the role of miR-155 in the pathogenesis of MS [74]. Junker et al. analyzed the profiles of miRNAs in active and inactive MS lesions. They showed that miR-155 was upregulated in active white matter lesions compared to healthy controls. In addition, the overexpression of miR-155 was related to the inhibition of the CD47 factor, which has the function of inhibiting macrophage activity. The reduction of CD47 promotes myelin phagocytosis processes [75]. MiR-155 expression was found to be significantly increased in CD14^+^ and CD68^+^ monocytes of MS patients and in in vitro experiments on M1 polarized macrophages and microglia. In addition, in both in vitro cell types, treatment with an miR-155 mimic showed an increase in the production of inflammatory cytokines, while treatment with a miR-155 inhibitor showed a reduction in the expression of these factors [76].

In vivo studies showed that miR-155 levels changed during myelin autoantigen response. The research was conducted on an EAE model and on lymph nodes further stimulated with myelin-oligodendrocyte glycoprotein (MOG 35–55). The results of the miRNAs expression profile showed an upregulation in the expression of 3 miRNAs, miR-21, miR-301a and miR-155 in CD4^+^ T cells. The inhibition of miR-155 caused a downregulation of the IFN-γ and an upregulation of IL-4 [77].

Murugaiyan et al. conducted a study on the MS animal model of EAE. A high miR-155 expression in CD4^+^ T cells of the spleen, lymph nodes and CNS was detected compared to controls. In addition, in miR-155^−/−^ mice a reduction in inflammation of the CNS and a delayed course of the disease were noted. Finally, they showed a reduction in the EAE clinical severity after anti-miR-155 treatment, when it was administered before and after the onset of symptoms. These results identify miR-155 as a target for a future therapeutic approach in the treatment of this disease [78].

Further research confirmed the association of miR-155 with the pathological states of the EAE and MS. For example, Zhang et al. observed that the expression of miR-155 is related to the severity of the disease and affects the expression of inflammatory T cells. In fact, the overexpression of miR-155 caused an increase in the number of Th1 and Th17 cells and a more severe condition of EAE, while miR-155 attenuation caused low levels of these cells [79]. MiR-155^−/−^ Th17 cells were defective in their capacity to cause EAE [80].

Among the many processes in which miR-155 is involved in the pathophysiology of MS, its role in the neuroinflammation of BBB has also been identified. In fact, BBB dysfunction and breakage is a typical feature of MS. Lopez-Ramirez et al. studied miR-155 activity on BBB permeability of individuals with MS and EAE mice. It has been shown that miR-155 upregulation increased the permeability of BBB by negatively regulating the elements of the cell–cell and cell–matrix adhesion pathways, such as annexin-2 (ANXA-2) and claudin-1 (CLDN-1), as well as cell-to-extracellular matrix (ECM) interactions, dedicator of cytokinesis 1 (DOCK-1) and syntenin-1 (SDCBP) [81].

Overall, the overexpression of miR-155 in patients with MS and EAE animal models has led to the investigation of this molecule as a potential biomarker to monitor treatment reactivity. In fact, some studies were carried out with the aim of determining miR-155 as circulating biomarker able to predict the clinical response to MS treatments. The levels of miR-155 were downregulated after treatment with dimethylfumarate (DMF) and natalizumab in MS patients and stimulated DMF-pretreated microglial cells [82,83,84].

Treatment with natalizumab reduced miR-155 expression levels in PBMC and monocytes isolated from MS patients, and a correlation was observed with a decrease in gene expression of IL-17, IFNγ and TNF-α [84]. 

MiR-155 levels were significantly reduced even after treatment with glatiramer acetate (GA); in addition, in this study, an analysis of miRNAs in urinary exosomes, plasma and spinal cord was performed in the pre-hearing, onset and peak phases of EAE. The expression of miR-155 was upregulated before the onset of the disease, suggesting its early involvement in the development of EAE [85].

### 4.3. MiR-155 in Other Neuroinflammatory Disorders

A pro-inflammatory role of miR-155 has also been observed in other neurological diseases. The summary of experimental studies on miR-155 in neuroinflammatory diseases, such as Parkinson’s disease (PD), amyotrophic lateral sclerosis, ischemic stroke is reported in Table 3.

#### 4.3.1. Parkinson’s Disease

Although to date there are few studies on the role of miRNAs in PD, some studies have investigated the expression of miR-155 in this pathology.

PD is a progressive neurodegenerative disease characterized by the loss of dopaminergic neurons in substantia nigra pars compacta (Snpc) and diffuse α-synuclein (α-syn) protein aggregates [101].

Numerous evidence indicates neuroinflammation as the primary mediator in PD pathogenesis and progression. An increase in proinflammatory cytokines, such as TNF, IL-1β, IL-6 and IFN-γ, and T-cell fertilization was observed. The inflammatory process contributes to neurodegeneration and determines the amplification of neuronal damage and cell death in PD models [102].

In one study, the expression of several miRNAs in PBMC of PD patients treated with L-dopa and healthy controls was analyzed [87]. Levodopa is the most used drug for treating PD, as most of the symptoms are caused by dopamine deficiency [103]. MiR-155, miR-26a, miR-146a and miR-132 have been selected, as they are the most widely studied in neurodegenerative diseases. The data showed an upregulation of miR-155 in patients with PD compared to the control group. In addition, a reduction in miR-155 expression was observed in patients with a higher levodopa dose, suggesting a possible modulatory role of this treatment on miR-155 levels [87]. Higher expression of miR-155 was also detected in extracellular vesicles derived from neurons of PD patients [88].

Some studies reported that DJ-1, an early onset autosomal recessive PD gene, inhibited the expression of pro-inflammatory factors in astrocytes and microglia treated with IFN-γ through inhibition of STAT1 and regulated inflammation by maintaining SOCS1 expression through regulation of miR-155 levels. The DJ-1 setting on SOCS1 was highlighted on microglia and astrocyte cultures derived from wild-type mouse brains (WT) and DJ-1-KO. IFN-γ significantly increased the expression of SOCS1 in microglia WT and astrocytes, but not in KO cells. In addition, the reduction of SOCS in DJ-1-KO cells seemed to be mediated by miR-155. In fact, IFN-γ increased miR-155 levels in DJ-1-KO cells but not in WT cells. In addition, treatment with a miR-155 inhibitor restored SOCS1 levels and reduced activation of STAT1 in DJ-1-KO cells [89]. 

The role of miR-155, in particular miR-155-5p form, has been investigated in PD animal models treated with rosmarinic acid (RA). In previous studies, it has been found that the use of RA reduced inflammatory responses and improved motor function. PD mice were treated with miR-155-5p agomir. Upregulation of miR-155-5p invalidated the effect of RA, causing microglia activation and increased inflammation, leading to a worsening of motor skills [90]. 

Thome et al. showed the overexpression of miR-155 in a PD in vivo model. In mice with full miR-155 deletion, a reduction of the proinflammatory response and the blockage of neurodegeneration induced by α-syn was noted. Treatment with a synthetic mimic of miR-155 restored the inflammatory response. These data suggest that miR-155 could be a potential therapeutic target for the regulation of inflammatory response in PD [91].

#### 4.3.2. Ischemic Stroke

Recent evidence suggests the involvement of miR-155 also in inflammatory processes in cerebral ischemia [92]. Ischemic stroke (IS) is characterized by a complex set of events that cause irreversible brain damage and is a major cause of death and disability. Inflammatory processes play an important role in tissue damage in ischemic stroke [104]. The ischemic event leads to the activation of microglia and astrocytes, resulting in the secretion of cytokines and chemokines. These inflammatory mediators cause an over-regulation of cell adhesion molecules on endothelial cells, allowing the infiltration of neutrophils, which, by secreting other cytokines, amplify the activation of glial cells. All of these processes lead to the death of neuronal cells and to the worsening of the damage [105].

The serum expression of miR-155 was evaluated in patients with acute IS. The increase in miR-155 expression was associated with a significant increase in JAK2/STAT3 expression in correlation with increased levels of TNF-α. It has been shown that these high levels of expression could trigger post-stroke cell inflammation and miR-155 could be used as a potential inflammatory biomarker for the acute phase of IS [93].

In order to investigate the potential role of miR-155 as biomarker in IS, levels of endothelial microvesicles (EMVs) carrying miRNAs were analyzed. EMVs may reflect the state of the endothelial cells involved in the pathogenesis of IS. EMV-miR-155 plasma levels increased significantly in acute and subacute stages of IS and were positively correlated with infarct volume. Data suggest EMV-miR-155 as a biomarker of IS [94].

In addition to potential biomarker characteristics, miR-155 could be a potential therapeutic target in the prevention and treatment of inflammatory damage after IS. In fact, miR-155 signaling was downregulated following treatments with substances that can reduce inflammatory processes [92,95,96]. A study evaluated the effects of the Gualou Guizhi decoction (GLGZD) treatment on neuronal damage induced by neuroinflammation mediated by miR-155. GLGZD has neuroprotective effects and improves damage caused by inflammatory processes induced by microglia. The results showed that pretreatment with GLGZD in HT22 and BV2 cells reduced miR-155signaling, resulting in a decrease of microglia activation and of the levels of TNF-α, IL6 and IFN-γ [95]. The effect of GLGZD was also evaluated on the MCAO rat model. The treatment inhibited upregulation of miR-155 with reduction of SOCS1 and levels of anti-inflammatory cytokines, resulting in improvements of neuronal deficits and motility [96].

In CD1 and C57/BL6 mice with MCAO the relationship between miR-155 and acetylbritannilactone (ABL), a potent anti-inflammatory substance used in the pharmacological treatment of IS, was studied. The results showed the inhibitory effect of ABL on gene expression mediated by miR-155. Inflammatory responses in ischemic brain tissue were modulated by miR-155 through regulation of the expression of TLR4/Myd88 and SOCS1 and treatment with ABL suppresses the expression of miR-155, suggesting a new therapeutic approach based on miR-155 for IS [92].

Inhibition of miR-155 in experimental models of IS led to a significant reduction in the production of pro-inflammatory cytokines and an improvement in infarct size and neuronal damage [97].

In C57BL/6-MCAO mice treated with miR-155 inhibitor, a significant reduction in the expression of pro-inflammatory cytokines and an altered expression of the target proteins of miR-155, such as SOCS-1 and SHIP-1, were observed. Based on these results, it appears that in vivo inhibition of miR-155 after mouse stroke changed the expression of the main cytokines and molecules associated with inflammation, which could affect the inflammatory process and tissue repair after experimental cerebral ischemia [97].

Caballero-Garrido et al., showed the effect of in vivo inhibition of miR-155 on brain recovery after ischemic stroke. The results obtained showed a 34% reduction of infarct size and a decrease of neuronal damage in the peri-infartual area of stroke after injection with a miR-155 inhibitor. A reduction in brain tissue damage and functional recovery were also observed [98].

#### 4.3.3. Amyotrophic Lateral Sclerosis

Treatment with an anti-miR-155 was also investigated in amyotrophic lateral sclerosis (ALS). Pro-inflammatory miRNAs were found to be differentially expressed in blood of patients with ALS and in the microglia of the SOD1 animal model. It was found that miR-155 regulated and reduced TGF-β1 expression [106].

ALS is a fatal neurodegenerative disease characterized by the loss of motor neurons in the spinal cord and brain, leading to stiffness, severe muscle weakness and death due to respiratory failure. ALS is not an inflammatory disease, but immune mechanisms seem to play an important role in the pathogenesis of the disease. In fact, several inflammatory responses were observed, and the activation of microglia and astrocytes was shown during the progression of the disease. Some evidence supports the involvement of these cells in neuronal death [107,108].

MiR-155 is also the most upregulated miRNA in microglia and spinal cord tissue of subjects with ALS and SOD1^G93A^ mice, and treatment with anti-miR-155 restored dysfunctional microglia, improved the disease and decreased the recruitment of monocytes in the spinal cord of SOD1 mice [99]. 

In the SOD1^G93A^ mouse model of ALS, inhibition of miR-155 led to increased expression of miR-155 target, such as SHIP-1, and slowing of disease progression with increased survival by 38%. In order to determine the therapeutic potential of miR-155 targeting in ALS, the variation of miR-155 expression was measured in both the rodent ALS model and the autoptic spinal cord tissue of ALS patients. MiR-155 was significantly increased 5-fold in mice and 2-fold in spinal cord tissue from familial and sporadic patient ALS. Therefore, the use of antisense oligonucleotides can lead to miRNAs inhibition throughout the brain and spinal cord, and miR-155 could be considered a promising new therapeutic target for human ALS [100].

#### 4.3.4. Traumatic Brain Injuries

Traumatic brain injuries (TBIs) represent one of the major causes of death and disability. In TBI brain damage is due to external forces and the main causes of TBI include vehicle and sport accidents and falls. TBI may include penetrating injuries, where a direct damage is caused to the brain parenchyma due to an object that breaches the skull and dura; in contrast, in closed-head injuries, the skull and dura are not damaged. On the bases of several parameters, such as consciousness, amnesia and neurological symptoms, TBIs can be divided into mild, moderate and severe [109]. The summary of experimental studies on miR-155 in TBI is reported in Table 4.

The upregulation of miR-155-5p at 24 h after TBI was found in the serum of TBI mice, but its level returned to baseline after 72 h, suggesting miR-155-5p’s role as an early TBI specific biomarker [110]. In addition, miR-155 levels were upregulated in mitochondria and cytosolic hippocampal fractions of TBI rats [111]. The miR-155 was significantly higher in the cytoplasm at 1 and 3 days post-TBI and returned to basal levels after 7 days. In mitochondria, miR-155 increased after 1 day [112].

In a controlled cortical impact (CCI) model of TBI, the miR-155 level was increased and mainly localized in neuronal nuclei. Interestingly, miR-155 KO mice showed a decreased expression of type I interferons IFNα and IFNβ, IFN regulatory factor 1 and CXCL10 (C-X-C Motif Chemokine Ligand 10) after TBI compared to wild-type (WT) mice. An unexpected result was that miR-155 KO mice showed elevated levels of the microglial marker Iba1, together with neuronal degeneration, indicating a neuroprotective role for miR-155 in TBI. This work suggested a role for miR-155 in the modulation of the IFN response and neurodegeneration in TBI [113]. 

TBI induced a neuroinflammatory response in parallel with increased miR-155 levels in the injured cortex, as well as in microglia and macrophages isolated from the injured cortex. In parallel to miR-155 increase, SOCS1 was reduced. The miR-155 inhibition reduced neuroinflammation and improved functional recovery. In particular, miR-155 antagomir administration after injury reduced neuroinflammatory markers at 1 day after injury, together with impairments in spatial working memory. Moreover, when miR-155 antagomir was administered, SOCS1 increased. Delayed miR-155 antagomir administration, 24 h post-injury for 6 days, attenuated neuroinflammatory markers 7 days post-injury and improved motor, but not cognitive, function. These data indicated that miR-155 can be considered a therapeutic target in TBI [114]. 

The striatum, even if it was not the primary site of impact, was shown to be highly susceptible to mitochondrial homeostasis disruptions in the delayed phase after cortical TBI. Interestingly, the reported mitochondrial dysregulation may be at least in part due to miRNAs. Indeed, an increase in miR-155 was found [115]. 

Interestingly, sex-specific alterations of several inflammatory miRNAs in CD11b^+^ cells of brain and bone marrow isolated from naïve and TBI mice were reported. Among these miRNAs, miR-155-5p was enriched in bone-marrow CD11b^+^ cells compared to brain. Specifically, the miR-155-5p level was elevated in male brain CD11b^+^ cells. TBI caused sex-specific responses. Indeed, after TBI, miR-155-5p increased in brain CD11b^+^ cells of both female and male mice, but female ones showed a greater induction after 3 h compared to male mice [116].

Kumar et al. evaluated the role of microparticles (MPs) that are members of the extracellular vesicle family in the transfer of pro-inflammatory mediators between brain immune cells and the systemic circulation, as a mechanism of inflammation propagation after TBI. After TBI, microglia-derived MPs were found to be released into the circulation. Circulating MPs isolated from TBI animals were able to activate BV-2 microglia in vitro. LPS treatment increased MP release from microglia in vitro and enhanced their content of pro-inflammatory mediators, including miR-155. Interestingly, enriched MPs derived from LPS-activated microglia in vitro or isolated from microglia/macrophage from the TBI brain were able to cause neuroinflammation in uninjured animals [117]. 

TBI patients can present intestinal mucosa dysfunction. The expression of miR-155 in the intestinal mucosa increased, while CLDN-1 decreased after 24 h of brain injury, causing an increase in intestinal mucosa permeability. These results indicated that miR-155 may be involved in intestinal mucosa dysfunction interfering with the expression of claudin1 [118]. 

Formononetin is a phytoestrogen belonging to flavonoid family, and its neuroprotective effects were tested in a TBI model. Formononetin pretreatment improved neurological scores, reduced brain edema and apoptosis in TBI rats. MiR-155 was found to be decreased, while BACH1 (BTB Domain And CNC Homolog 1) increased in the TBI model; however, formononetin pretreatment increased the expression levels of miR-155 and HO-1, while it reduced BACH1 in TBI rats [119].

Propofol is an anesthetic agent that showed neuroprotective effect in TBI models. Propofol in LPS-activated BV2 microglia reduced inflammatory mediators and the expression of miR-155. Knockdown of miR-155 reduced the anti-inflammatory effect of propofol. miR-155 was also confirmed as a negative regulator of SOCS1 expression. Then the results suggested that propofol can suppress the neuroinflammatory response of microglia to LPS modulating the miR-155/SOCS1 pathway [120].

#### 4.3.5. Neuroinflammation Induced by Viral Infections

MiR-155 is also involved in the regulation of neuroinflammation induced by viral infections. Several studies have highlighted several regulatory mechanisms with which miR-155 controls immune responses in neurological-virus-induced disease models [121]. 

The summary of experimental studies on miR-155 in neuroinflammation induced by viral infections is reported in Table 5.

Herpes simplex virus (HSV) infection may cause severe herpes simplex encephalitis (HSE). It was reported that, during acute HSV type 1 (HSV-1) encephalitis in mice, miR-155 was upregulated in brain tissue. Specifically, it was suggested to be upregulated in immune cells that infiltrate the brain [122]. Moreover, mice with a deficiency of miR-155 were highly susceptible to HSE, given that the majority of animals developed HSE after ocular infection with HSV-1. The miR-155 KO animals were also more susceptible to develop zosteriform lesions, indicating viral replication and dissemination in the nervous system. This effect may be due to the fact that miR-155 KO animals showed diminished CD8 T-cell responses. MiR-155 expression may influence susceptibility of the nervous system to virus infection [123].

The role of miR-155 was also evaluated in a model of neuroinflammation induced by the inoculation of the neurotropic JHM strain of mouse hepatitis virus (JHMV) into the CNS of mice. JHMV-infected miR-155^−/−^ mice presented a delayed disease onset, even if clinical disease was sustained, in association with an elevated morbidity and mortality rate compared to WT mice. Moreover, JHMV-infected miR-155^−/−^ mice showed high viral titers. JHMV-infected miR-155^−/−^ mice showed reduced CD8^+^ T-cell responses. IFN-γ responses were impaired in miR-155^−/−^ virus-specific CD4^+^ T cells. There were differences in macrophage CNS infiltration only very early during infection between WT and miR-155^−/−^ JHMV-infected mice. Moreover, the severity of demyelination was similar between WT and miR-155^−/−^ JHMV-infected mice. These results suggested that miR-155 has a role in host defense in viral-induced encephalomyelitis, inducing antiviral T-cell responses [124].

West Nile virus (WNV) is a member of *Flavivirus* that is known to cause viral encephalitis in humans. During WNV infection, miR-155 is upregulated in mice brains, and it has a critical role in restricting its pathogenesis. Indeed, miR-155 KO mice showed elevated morbidity and mortality rates after infection with both lethal and non-lethal strains. In parallel, miR-155^−/−^ mice showed a high virus titer in the serum and the brain. IFN-α in the serum and brains was higher in miR-155^−/−^ mice, while the levels of antiviral interleukins were reduced. Moreover, in vitro, primary mouse MEFs and BMDMs cells lacking miR-155 showed higher virus titers compared to WT cells. On the contrary, upregulation of miR-155 in human neuronal cells lowered WNV replication. These results indicated that miR-155 restricted WNV production and protected against lethal WNV infection in mice [125].

Japanese encephalitis virus (JEV) is a neurotropic virus that is able to infect microglia. An increase of miR-155 was found in microglia BV-2 cells and in the primary microglial culture of mice BALB/c after JEV infection. The targeting of SHIP1 by miR-155 was also evaluated. Inhibition of SHIP1 resulted in an increase of IFN-β and proinflammatory cytokines through activation of TANK-binding kinase 1 (TBK-1). Treatment with anti-miR-155 showed a reduction in IFN-β, TNF-α and IL-6, resulting in a lower inflammatory response [126]. 

Moreover, it was found that miR-155 expression decreased 6 h post-infection in human microglial cells infected with JEV, followed by an increase at 48 h. Overexpression of miR-155 was accompanied by a reduction of viral replication. Moreover, the upregulation of miR-155 reverted the JEV-mediated induction of interferon regulatory factor 8 (IRF8) and complement factor H (CFH). MiR-155 overexpression also increased levels of CD45, a negative regulator of microglia activation, while it reduced phosphorylated Signal Transducers and Activators of Transcription (p-STAT1) and cytokines. Then miR-155 in microglial cells may modulate JEV-induced innate immune gene expression and exert a positive role in limiting JEV replication in human microglial cells [127].

Zika virus (ZIKV) infections may cause a wide spectrum of neurological diseases. The infection induced a major downregulation of miRNAs; only a few miRNAs were upregulated. Interestingly, miR-155 was among the upregulated miRNAs. On the contrary, antiviral, inflammatory and apoptotic genes were upregulated [128].

Another work evidenced that the miR-155-mediated regulation of SOCS1 expression was superfluous for the acute expansion of CD8^+^ T cells, but was indispensable for the maintenance of the antiviral T-cell response during chronic infection [129].

Human immunodeficiency virus (HIV) is known to infect microglia and macrophages, but astrocytes are restricted to HIV infection. The restriction factor sterile alpha motif and histidine/aspartic acid domain-containing protein 1 (SAMHD1) is known to restrict HIV infection in resting CD4^+^ T cells and in monocyte-derived dendritic cells, and researchers evaluated if it could also be responsible for the HIV restriction in astrocytes. HIV replication reactivation was associated to SAMHD1 decrease. SAMHD1 expression increased and miR-155 and miR-181a expression decreased in astrocytes compared to microglia-infected cells, indicating that these miRNAs regulated SAMHD1 expression. Overexpression of these miRNAs increased viral replication in the astrocytes, modulating SAMHD1 [130].

## 5. Conclusions

**Figure 1:**
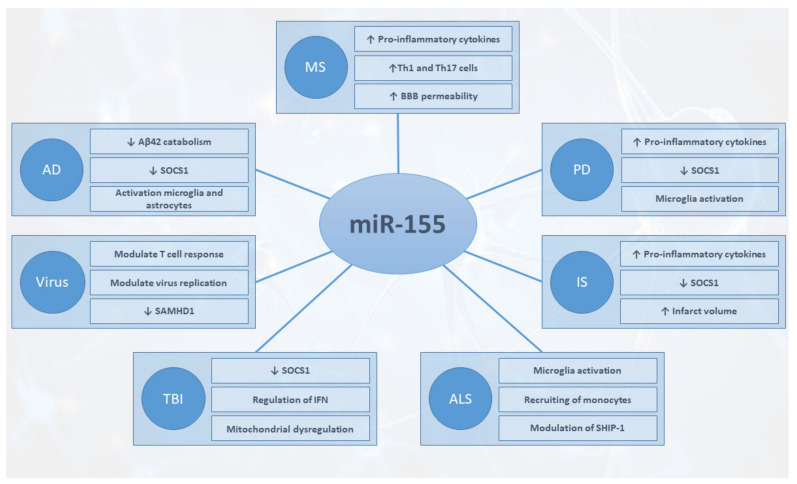
Major mechanisms affected by miR-155 in the different pathologies. AD, Alzheimer’s disease; ALS, amyotrophic lateral sclerosis; IS, ischemic stroke; MS, multiple sclerosis; PD, Parkinson’s disease; TBI, traumatic brain injury.

MiRNAs play a key role in the control of many processes, and their dysregulation is associated with the onset and progression of several diseases, including neurodegenerative and neuroinflammatory diseases. The different mechanisms affected by miR-155 in the setting of the different pathologies are reported in Figure 1.

MiR-155 is involved in neuroinflammation and, in particular, in the activation of microglia and astrocytes and, consequently, in the production of pro-inflammatory cytokines. Numerous studies support its involvement in many neurological diseases, and it could be considered a candidate biomarker. MiR-155 also has a role in neuroinflammation due to viral infection, influencing also viral replication. However, miR-155 may be useful to monitor the effectiveness of treatments, but it also represents a potential therapeutic target. Indeed, some agents that inhibit miR-155 lead to a reduction of inflammation, apoptosis and histopathological alterations in AD, MS, PD, IS and ALS models. It could also be a therapeutic target for TBI. Interestingly, the American company miRagen Therapeutics, has already developed a drug (Cobomarsen) designed to inhibit the activity of miR-155 in patients with T-cell cutaneous lymphoma, and a preclinical trial was performed for the drug MRG-107, targeted at miR-155 designed for the treatment of ALS [131]. A greater understanding and exploration of the mechanisms of miR-155 can contribute to the initiation of further studies on the application of these molecules in clinical practice.

## Figures and Tables

**Table 1:** Summary of studies reported on miR-155 in AD.

Model/Cell Type	Expression of MiR-155	Results	References
LPS-stimulated microglia cells	Up	<Aβ42 catabolism	[57]
BDMs and MDMs of AD	Up	activation status M1	[58]
ECF and CSF of AD patients HNG cells	Up	induction inflammatory gene expression	[59]
HNG cells	Up	CAPE and CAY10512 agents: <miR-155	[60]
APPtg and TAUtg mice	Up	AD genes correlation	[61]
SAMP8 mice	Up	AD genes correlation	[62]
Model 3xTg AD N9 microglia cells Astrocyte	Up	activation of microglia and astrocytes <SOCS-1 <MiR-155 after treatment with siRNA c-Jun	[63]
ApoE3-5xFAD mice	Up	Curcumin: <miR-155	[64]
Wistar rats/LPS	Up	H2S and MSC: <miR-155	[65]
Rat hippocampus AD	Up	Anti-miR-155: <IL-1β, IL-6, TNF-α <cognitive impairment	[66]

AD, Alzheimer’s disease; Aβ, beta-amyloid peptide; IL, interleukin; TNF, tumor necrosis factor; BDMs, blood-derived monocytes; MDMs, monocyte-derived macrophages; LPS, lipopolysaccharide; ECF, extracellular fluid; CSF, cerebrospinal fluid; HNG, human neuronal–glial; CAPE, caffeic acid phenethyl ester; CAY10512, 1-fluoro-2-[2-(4-methoxyphenyl)-ethenyl]-benzene; SOCS-1, Suppressor of Cytokine Signaling; H2S, hydrogen sulfide; MSC, mesenchymal stem cells.

**Table 2:** Summary of studies reported on miR-155 in MS.

Model/Cell Type	Expression of MiR-155	Results	References
serum and plasma of MS patients	Up	-	[73]
PBMC of MS patients	Up	-	[74]
lesions of MS	Up	inhibition CD47	[75]
CD14^+^ and CD68^+^ of MS patients	Up	anti-miR-155: <inflammatory cytokines	[76]
EAE	Up	Anti-miR-155: <IFN-γ > IL-4	[77]
EAE	Up	anti-miR-155: <clinical severity	[78]
EAE MS patients	Up	>Th1 and Th17 cells	[79]
EAE miR-155^−/−^ cells	-	<Th17 cells	[80]
EAE MS patients	Up	>BBB permeability	[81]
MS patients	Up	DMF treatment: <miR-55	[82,83]
MS patients	Up	Natalizumab treatment: <miR-155	[84]
MS patients	Up	GA treatment: <miR-155	[85]

MS, multiple sclerosis; PBMC, peripheral blood mononuclear cells; MOG, myelin-oligodendrocyte glycoprotein; IFN, interferon; IL, interleukin; EAE, experimental autoimmune encephalomyelitis; BBB, blood–brain barrier; DMF, dimethylfumarate; GA, glatiramer acetate.

**Table 3:** Summary of experimental studies on miR-155 in PD, ALS and IS.

Disorder	Model/Cell Type	Expression of MiR-155	Results	References
PD	PBMC of PD patients	up	-	[87]
	NDsEV	up	-	[88]
	Microglia/astrocyte	up	<SOCS	[89]
	PD’s model	-	miR-155-5p agomir: <efficacy RA >inflammation microglia activation	[90]
	PD’s model MiR-155 ^−/−^	-	<proinflammatory signaling Blockage neurodegeneration	[91]
IS	CD1 and C57/BL6 mice with MCAO	up	Correlation miR-155/ABL	[92]
	Serum of IS patients	up	>JAK2/STAT3 >TNF-α	[93]
	EMVs	up	>infarct volume	[94]
	HT22 and BV2 cells	up	GLGZD treatment: <miR-155 <TNF-α, IL6 and IFN-γ	[95]
	MCAO rat	up	GLGZD treatment: <miR-155 <SOCS1	[96]
	MCAO C57BL/6 mice	up	anti-miR-155: <pro-inflammatory cytokines	[97]
	MCAO C57BL/6 mice	up	anti-miR-155: <damage	[98]
ALS	mice SOD1^G93A^	up	anti-miR-155: restored dysfunctional microglia	[99]
	mice SOD1^G93A^	up	anti-miR-155: >38% survival	[100]

PD, Parkinson’s disease; IS, ischemic ictus; ALS, amyotrophic lateral sclerosis; PBMC, peripheral blood mononuclear cells; NDsEV, extracellular vesicles derived from neurons; SOCS, Suppressor of Cytokine Signaling; JAK, Janus tyrosine kinase; STAT, signal transducer and activator of transcription; IFN, interferon; IL, interleukin; TNF-α, tumor necrosis factor; RA, rosmarinic acid; ABL, acetylbritannilactone; EMVs, endothelial microvesicles; GLGZD, Gualou Guizhi decoction; MCAO, middle cerebral artery occlusion.

**Table 4:** Summary of experimental studies on miR-155 in TBI.

Model/Cell Type	Expression of MiR-155	Results	References
Serum of TBI mice	Up	-	[110]
Mitochondria and cytosolic hippocampal fractions of TBI rats	Up	-	[111]
Cytoplasm and mitochondria	Up	-	[112]
CCI model induced in C57BL/6 mice and MiR-155 KO	Up	MiR-155 KO: <type I interferons >Iba1	[113]
CCI C57Bl/6J mice	Up	<SOCS1 anti-miR-155: <neuroinflammation	[114]
CCI Long Evans rats	Up	Mitochondrial dysfunction	[115]
CD11b^+^ cells	Up	-	[116]
CCI C57BL/6 mice; BV2 cells treated with LPS	Up	Microglia isolated MP contained miR-155 and propagate inflammation	[117]
Intestinal mucosa TBI patients	Up	<CLDN-1 intestinal mucosa dysfunction	[118]
TBI Wistar rats	-	Formononetin increased miR-155	[119]
LPS-treated BV2 cells	-	Propofol reduced miR-155	[120]

CCI, controlled cortical impact; SOCS, Suppressor of Cytokine Signaling; CLDN-1, Claudin-1; MPs, Microparticles; TBI, traumatic brain injury.

**Table 5:** Summary of experimental studies on miR-155 in neuroinflammation associated to viral infections.

Model/Cell Type	Expression of MiR-155	Results	References
HSV-1 mice	Up	-	[122]
miR-155^−/−^ mice	-	>susceptible to HSE	[123]
JHMV miR-155^−/−^ mice	Up -	- elevated morbidity and mortality rate <T CD8^+^	[124]
WNV miR-155^−/−^ mice	Up -	- miR-155^−/−^ mice: >high virus titer >IFN-α	[125]
BV-2 and mice BALB/c with JEV	Up	<SHIP1 anti-miR-155: <IFN-β, pro-inflammatory cytokines	[126]
CHME3 cells infected with JEV	Up	<viral replication >CD45 <p-STAT1	[127]
ZIKV	Up	-	[128]
LCMV-infected mice	-	Regulation of SOCS1	[129]
HIV-infected astrocytes	-	Regulation of SAMHD1	[130]

HSE, Herpes simplex encephalitis; SOCS, Suppressor of Cytokine Signaling; IFN, interferon; SHIP1, SH2 Domain-Containing Inositol 5′-Phosphatase1; p-STAT1, Phosphorylated-Signal Transducers and Activators of Transcription; HSV, herpes simplex virus; JHMV, JHMV: strain of mouse hepatitis virus; WNV, West Nile virus; JEV, Japanese encephalitis virus; ZIKV, Zika virus; HIV, Human immunodeficiency virus; SAMHD1, Sterile alpha motif and histidine/aspartic acid domain-containing protein 1.

## Abbreviations

ABL	Acetylbritannilactone
AD	Alzheimer’s disease
AGO-2	Argonaute 2
ALS	Amyotrophic lateral sclerosis;
ANXA-2	Annexin-2
Aβ	Beta-amyloid peptide
BACE1	β-site APP cleaving enzyme
BACH1	BTB Domain And CNC Homolog 1
BBB	Blood–brain barrier
BDMs	Blood-derived monocytes
CAPE	Caffeic acid phenethyl ester
CAY10512	1-fluoro-2-[2-(4-methoxyphenyl)-ethenyl]-benzene
CFH	Complement factor H
CLDN-1	Claudin-1
CNS	Central nervous system
CSF	Cerebrospinal fluid
CXCL10	C-X-C Motif Chemokine Ligand 10
DGCR8	DiGeorge Syndrome Critical Region Gene 8
DMF	Dimethylfumarate
DOCK-1	Dedicator of cytokinesis 1
EAE	Experimental autoimmune encephalomyelitis
ECF	Extracellular fluid
ECM	Extracellular matrix
EMVs	Endothelial microvesicles
GA	Glatiramer acetate
GLGZD	GualouGuizhi decoction
H2S	Hydrogen sulfide
HIV	Human immunodeficiency virus
HNG	Human neuronal–glial
HSE	Herpes simplex encephalitis
HSV	Herpes simplex virus
JHMV	strain of mouse hepatitis virus
IFN	Interferon
IL	Interleukin
IL13Rα1	Interleukin 13 receptor alpha 1
iNOS	Inducible nitric oxide synthase
IRF8	Interferon regulatory factor 8
IS	Ischemic ictus
JAK	Janus tyrosine kinase
JEV	Japanese encephalitis virus
KO	Knockout
LPS	Lipopolysaccharide
M1	Pro-inflammatoryphenotype
M2	Anti-inflammatoryphenotype
MDMs	Monocyte-derived macrophages
MMP-9	Matrix metalloproteinase
MOG	Myelin-oligodendrocyte glycoprotein
MPs	Microparticles
MS	Multiple sclerosis
MSC	Mesenchymal stem cells
NDsEV	Extracellular vesicles derived from
NF-κB	Nuclear factor κB
NO	Nitric oxide
NVU	Neurovascular unit
p53	Tumor protein P53
PBMC	Peripheral blood mononuclear cells
PD	Parkinson’s disease
PI3K	Phosphoinositide 3-kinase
PPMS	Primary progressive multiple sclerosis
RA	Rosmarinic acid
RISC	RNA-Induced Silencing Complex
ROS	Reactive oxygen species
RRMS	Relapsing-remitting multiple sclerosis
SAMHD1	Sterile alpha motif and histidine/aspartic acid domain-containing protein 1
SDCBP	Syntenin-1
SHIP1	SH2 Domain-Containing Inositol 5′-Phosphatase1
SNPs	Single nucleotide polymorphisms
SOCS1	Suppressor of cytokine signaling
SPMS	Secondary progressive multiple sclerosis
STAT	Signal transducer and activator of transcription
p-STAT1	Phosphorylated-Signal Transducers and Activators of Transcription
TBI	Traumatic brain injury
TBK-1	TANK-binding kinase 1
TGF-β	Transforming Growth Factor β
TLRs	Toll-like receptors
TNF-α	Tumor necrosis factor
TRBP	TAR RNA binding protein
WNV	West Nile virus
WT	Wild-type
α-syn	α-synuclein
ZIKV	Zika virus
